# Multisensory perception and action in painting: science, creativity, and technology

**DOI:** 10.3389/fpsyg.2025.1630107

**Published:** 2025-08-21

**Authors:** Monica Gori, Giulio Sandini

**Affiliations:** ^1^Istituto Italiano di Tecnologia – Unit for Visually Impaired People (U-VIP), Genova, Italy; ^2^Istituto Italiano di Tecnologia – Robotics, Brain and Cognitive Sciences (RBCS), Genova, Italy

**Keywords:** art, perception, action, multisensoriality, art—decorative arts

## Abstract

Painting comes from the desire to create through movement and color a non-existent image, making it real. To create a new pictorial composition, a close harmony between the creative process and the motor act is necessary. The technique represents the ability to generate motor actions and to interpret the “language” of colors and shapes. Creativity is the ability, through imaginative, cognitive, and perceptive sensory skills, to transfer creative work on canvas. The action itself is, therefore, a disappearing ghost of creativity; leaving the result of the pictorial work. In this article, starting from the reflections of those who preceded us, we will discuss the intersection between neuroscience and art by discussing action and multisensory perception in painting. We propose an interdisciplinary link between art, neuroscience, and technology that can open up new research directions and artistic methodologies. The final goal of our perspective is to highlight the process behind the creation of a painting highlighting how action, multisensory perception and new technologies interact in the creative process.

## 1 Introduction

Art and perception are closely linked, and all forms of art are expressed through the body’s movement. Some forms of art are perceived in a dynamically observable way, like dance, while others are usually perceived after the motor act has been completed, like painting. In this case the artistic expression arises from the desire to reproduce a visual scene or create a new image through movement and a chromatic composition, to represent something that cannot be seen or that is reproduced. Through the brushstroke, the artist creates a new visual image that stimulate a new percept, while the action itself disappears forever, leaving behind traces that express the artistic content and characterize the expressive technique. Creativity, that is defined as the ability to produce work that is both novel and appropriate (Botella and Lubart, 2015). The creative process involves a series of thoughts and actions that lead to these original and appropriate productions and are therefore directly associated with my many perceptual, motor and cognitive human skills.

Many authors in the past have discussed and studied the relationship between perception and art. For example, Maffei and Fiorentini, in their book Art and Perception, specifically address how the human eye comes to perceive a work of art (Maffei and Fiorentini, 2008). Eric Kandel (Nobel Prize winner for Medicine for his studies on memory) also describes the relationship between mind, brain, and art (Kandel, 2012), and V. S. Ramachandran explores the uniqueness of human nature from a neuroscientific perspective, linking synesthesia, creativity, and esthetics (Ramachandran, 2011).

In this article, building on the reflections of those who have preceded us on this topic, we discuss the intersection between neuroscience and art, presenting two aspects that are closely linked to the concept of art and perception: namely the role of multisensory perception and the connection between perception, artistic content, and action with special emphasis on visual art. In doing so, we discuss about the new methodologies and technologies related to these themes and the relationship between artistic creativity and sensory, motor, and cognitive abilities. The final goal of the perspective is to describe from a neuroscientific point of view the creative process through which the idea becomes “concrete.” In the process multisensory perception, actions, and technology come into play. We propose that, to study the creative process, it is necessary to consider all these aspects even if, in the “final product,” not all of them appear explicitly (such as the painter’s movement which, in the end, leaves “only” something static to look at). Through the exploration of these themes, we aim to show the potential for interdisciplinary work between art, neuroscience, and engineering, opening new directions for research on human creativity and artistic methodologies.

### 1.1 Multisensory perception and the connection between perception and action

Our sensory perception is the window with the world around us. We wake up in the morning and open our eyes. Thanks to our sight, we can immediately tell if the sun has already risen. Through hearing, we can perceive if someone else is already awake in the house, and through touch, we can grab the blankets and uncover ourselves to get out of bed. With our sense of smell, we can perceive the aroma of coffee in the morning. The concept of “morning” is a multisensory percept.

These simple actions are the result of complex processes of proprioceptive and exteroceptive sensory information processing mediated through experience and expectations. When a signal from outside comes into contact with our sensory receptors, our brain constructs a percept of the event and produces a response. Perceptual signals are incredibly rich and evocative. From the technological point of view for example, the human eye can see three basic colors but is able to interpret 7 million variations of colors (Geldard, 1972). These variations are created by combining the three primary colors: red, green, and blue. Hearing is never inactive, even when we sleep, and the human olfactory system consists of many millions of olfactory sensory neurons (Keller and Vosshall, 2004). The complexity in the analysis of these signals allows us to have a vast perceptual richness and to live in an ever-changing and multifaceted environment, characterized by both the individual sensory modalities and the ability to integrate them in a multisensory way, where the action plays a fundamental role (for example, the aroma, taste, and temperature of coffee integrate with the weight of the cup and the action of bringing it to the mouth to sip its contents). All of this happens even though each sensory system has its own timing in analyzing signals. Visual and auditory signals, for example, follow two different pathways and consequently have different “processing times.” Nonetheless, our brain provides us with a synchronized perception of the two signals in space and time, and this integration at the cerebral level enhances our sensory experience of the world (Calvert et al., 2004). In fact, when sensory signals of different modalities are active at the same time, the brain improves its perception in terms of precision, accuracy, and reaction time through processes of multisensory integration (Gori et al., 2011), making our interaction with the surrounding world more effective.

In the field of experimental psychology and cognitive and computational neuroscience, these processes are described by mathematical models that allow us to quantify the sensory advantage obtained from multisensory integration (Ernst and Banks, 2002). In this context, the role of action is important not only for controlling interaction with the environment but also for “synchronizing” the individual sensory modalities into a unified percept. Action and perception must necessarily coexist and constitute an interdependent pair in the context of cognitive neuroscience. Studies on the human brain highlight the presence of interconnected neural circuits and the development of this bidirectional relationship between perception and action. From infancy to adulthood, movements skills improve and become more precise and accurate. In the early years of life, movements and sensory modalities are crucial; especially trough the haptic process for the refinement of multisensory integration processes (Gori et al., 2008). The ability to integrate action and perception is fundamental because it allows us to optimally connect with the surrounding world, perform goal-directed actions, and anticipate (imagine) not only the motor objective of the action but also the sensations that will be perceived before the action itself begins (Schum et al., 2011; Hofsten and Fazel-Zandy, 1984). The multisensory integration of sensory signals and the communication of such information into motor behaviors are fundamental processes for understanding human cognition and constitute a crucial area of research for comprehending the complexity of mind–body interactions.

## 2 Painting, movement, and multisensoriality

### 2.1 Multisensoriality and painting

Vision is often considered the primary sense involved in painting, but using a paintbrush or another paint-application device (and even painting by naked hands) fundamentally and profoundly stimulate painter’s tactile and haptic perception system and interact with visual perception during painting. Multisensory painting, therefore, a fundamental an artistic experience, extending the interaction between the artwork, the artist, (and sometimes the observer beyond) just vision. The most used sensory modality paired with sight is haptic perception, which originates from action and refers to the integration of touch and proprioception. This is stimulated by the interaction between the tool manipulated by the artist (e.g., the brush), the support (e.g., the canvas), and the movement of the hand, which includes the spatial trajectory, orientation and the force exerted, i.e., the kinematics and dynamics of the motor act. In painting, the complexity of these (inter)actions is transferred to the mark left on the canvas but is irretrievably lost. Painters can exploit haptic sensibility to create particular textures on the canvas by transposing mental images into motor programs through a “mental simulation” phase that precedes the execution itself (Jeannerod and Jacob, 2005; Jeannerod, 2001). Is the motor act the creative part of the stroke or the action is “just” trying to reproduce what has been visualized mentally? Most probably a combination of both with the added variable of the painting “material” used.

For example, Vincent van Gogh often used thick brushes and impastos to create rough and palpable surfaces that invite the viewer to mentally touch his art, activating a mechanism of “reverse imagination” that takes the viewer from the mark imprinted on the canvas back to the movement that generated it. In the case of Vincent van Gogh’s works, the hidden haptic sensation that emerges when observing the painting represents one of the distinctive features of his work. This is an example where multisensory integration works “in reverse,” generating tactile and motor sensations from observing the surface of the painting. Although the enjoyment of the artwork occurs primarily in the realm of explicit iconic representation, the hidden motor/proprioceptive representation remains one of the main components of the artistic creation, the interpretation of which also depends on the observer’s personal multisensory and motor experience. It depends on how personally and directly familiar the observer is with the technique. This assertion is based on at least two observations derived from the study of our nervous system: the first is that our ability to interpret others’ actions (i.e., to anticipate their effects) develops in parallel with our ability to perform those same actions (Falck-Ytter et al., 2006; Von Hofsten, 2007); the second is that the areas of our brain dedicated to execution and planning (“action emulation,” “motor imagery”) are shared (Jeannerod, 2001; Ptak et al., 2017).

Another example is Georgia O’Keeffe, who emphasized the tactility of the objects depicted, leading viewers to reflect on the tactile sensation through the shapes and pictorial patterns. Finally, Wolfgang Laib is known for his artworks made with natural materials such as pollen, rice, and wax, thereby involving the sense of touch, smell, and sight, creating multisensory meditation spaces that, although primarily stimulated through sight, have the power to stimulate our tactile and motor imagination. This is a form of synesthesia, a sensory/perceptual phenomenon indicating a “cross-contamination” of the senses in perception (Ward and Mattingley, 2006), which other artists have exploited in the interaction between sight and hearing. The most famous example is Wassily Kandinsky, a 20th-century abstract painter known for trying to capture the essence of music through painting. Kandinsky created artworks inspired by musical compositions and claimed that color and form could evoke emotions similar to those created by music. His works are often considered an example of artistic synesthesia, where different senses overlap. Kandinsky stated: “Music does not need to borrow external forms to use in its language. Painting, however, is today bound to forms drawn from nature. Just as music does not necessarily have words to be beautiful but can be composed solely of sounds and rhythms, so can painting be composed solely of colors, lines, and forms. Color is the key, the eye is the hammer, and the soul is the piano with many strings.” On the other hand, the performance and enjoyment of music also have an evident motor content which, although more evident and important in ensemble performances (Badino et al., 2014), significantly contributes to the “sensations” conveyed by a piece (for example, the emotional content of a piece through the gestures of the violinist).

In the work titled “Not Just Seeing, but Also Feeling Art: Mid-Air Haptic Experiences Integrated in a Multisensory Art Exhibition” (Ablart et al., 2017) the authors ask whether mid-air haptic technology integrated with sound can enhance the experience of visual art. The results show how this new mid-air technology can make art more engaging and emotionally stimulating, particularly abstract art, which is often open to interpretation. The interaction between visual and gustatory experience has also been reported. In the article “A Taste of Kandinsky,” the authors evaluated the influence of the artistic visual presentation of food on the culinary experience (Michel et al., 2014) demonstrating that the artistic visual presentation of food enhances the quality of gustatory perception when eating it. This form of multisensory perception characterizes the artistic expression of the cuisine of various countries, such as Japanese cuisine, and justifies the care taken in the “plating” phase of foods (in this case, visual perception remains subordinate to smell and taste). These examples of multisensory artistic expression demonstrate how art can engage a wide range of human senses.

### 2.2 Movement and painting

During painting, variations in pressure, the amplitude of the gesture, and the speed with which the tool is moved (as well as the nature of the tool itself) contribute to creating unique lines, shapes, and textures in the artwork. The kinematics and dynamics of the artist’s movement are thus at the origin of the artistic result. During the creation of a pictorial artwork, and more generally in the figurative arts, the artist’s iconic imagination is translated into motor imagination, as if the brush became an extension of their body, breaking down thoughts and emotions into movements that materialize on the canvas. Abstract concepts take form and are defined by the movements themselves. For example, broad and fluid movements can convey a sense of peace, controlled movements can emphasize details or transmit a calmer, more contemplative atmosphere, while short and sharp strokes can evoke a sense of dynamism and energy (similarly to how a musician’s gestures transmit, amplify, and detail the emotional content of music). These emotional aspects can be appreciated by the observer only by putting themselves in the artist’s shoes and trying to imagine the action that generated them, in a sort of reverse process stimulated by the ability to empathize with others—a characteristic of human beings that has been studied and described in the neuroscience of “mirror neurons” (Rizzolatti and Craighero, 2004) and about social cognition (Sciutti et al., 2012; Gallese et al., 2004) and that, very aptly, is referred to as a “resonant phenomenon” describing a situation in which two person are not only “seeing” each other but they are feeling like moving synchronously.

Except from filmed recordings that nonetheless capture only the kinematic aspects of movement during the creation of paintings, it is rarely possible to witness the motor phase of creative painting expression, which represents a fundamental, albeit hidden, creative physical aspect of painting. Over the years, it has played a very significant, yet ghostly, role. An significant example is the form of painting called gestural art which is characterized by the explicit use of free, sweeping gestures to apply paint, like Jackson Pollock’s action painting, where the impetuous and spontaneous movement of the arm and body determines the final composition of the work, and the creative gesture—the physical act of painting—becomes an integral part of the artwork itself and only those actually present during its creation can fully appreciate the artistic performance Conversely, precision and finesse of stroke can be distinctive in the works of artists such as Leonardo da Vinci or Albrecht Dürer. Ultimately, painting and movement are interconnected in a continuous dialog, where the artist explores and captures the dynamic essence of life through the visual medium of painting. Some artists are particularly skilled at “freezing” movement in such an intuitive way that they allow us to perceive motion even within a still image. This is yet another form of artistic expression where the artist does not show their own movements but instead creates the illusion of motion. Through techniques like blurred lines, unique perspectives, and the portrayal of multiple phases of an action within a single frame, artists can make the viewer experience movement as if it were alive. This approach is often seen in movements such as Futurism and Cubism, where movement is not just represented but becomes a core element of the composition.

Speaking about actions and painting we should consider the embodied approaches of cognition, that emphasize the crucial role of the body and its interactions with the environment in shaping cognitive processes (i.e., Dietrich and Haider, 2015). The artist’s embodied experience contains the spatial and temporal dimensions of the artistic impulse for the artistic process (Choi and DiPaola, 2013). Action and embodied cognition are strictly related. However, in this work we argue that the explicit relation between perceptual, multisensory and action kinematics at psychophysical and neurophysiological level should be better investigated by understanding how neuroscientific basic sensory-motor inputs relate with the embodied expression. Namely, we believe that the multisensory, gestural and technological components of art and their relationship with embodied creativity is more than the visual experience of the image and this interaction should be better investigated.

## 3 Technologies and methodologies: movement and multisensoriality

The focus of this work is on the role that multisensoriality and movement play in figurative artistic expression and to show how recent findings in computational neuroscience and the availability of tools for the quantitative analysis of human sensory, motor, and cognitive abilities can allow us to study the processes underlying the creation and enjoyment of artifacts and artworks. This goes beyond the apparent “final result” and includes the hidden action that produced them. The interest in studying the hidden role of action in the production of artwork stems from the curiosity to understand how our “brain” (in a broad sense) transforms the idea of artwork into the “motor program” necessary to realize it and the role of multi sensoriality in the creative process and the enjoyment of an artwork.

Another example of multisensory and motor integration that falls outside the scope focused on the “figurative” arts in this article concerns the so-called “sonification of movement” (Kolykhalova et al., 2016), in which an artist’s movement (for example, a dancer) is measured with external or wearable sensors and transformed into sounds and/or other sensory stimuli. In this case, movement is an integral part of the explicit artistic expression, unlike the painter’s movement, of which no explicit trace remains.

Perhaps it is precisely due to this absence of a motor trace that the study of the neuroscientific foundations of figurative art is focused on eidetic aspects, unlike studies dedicated, for example, to the parallel between neuroscience and performative art forms (for a recent analysis of these studies, see (Morasso and Morasso, 2021), co-written by a movement neuroscientist and a dancer/choreographer).

### 3.1 Technologies and methodologies: multisensoriality, movement, and painting

Given the variety and importance of artistic forms in which the artwork does not explicitly express the artist’s movement and the availability of devices for the quantitative measurement of human movement and the study of multisensory integration, the current century has opened up new technological opportunities and methodological approaches to studying painting. This applies not only from the observer’s perspective but also during the creative phase when motor actions, in its kinematic and dynamic aspects, guide the integration of visual perceptual aspects with haptic (the applied force and tactile sensation) and acoustic ones (the hammer’s strike, the burin’s scraping, the sound of the spatula spreading color).

Regarding multisensorial processing, Bayesian models suggest that different sensory modalities can have varying weights in different contexts (Gori et al., 2023) and in different individuals (Zanchi et al., 2022). This suggests that painting is perceived differently by all observers. For this reason, multisensory works, for example, visual and tactile ones, could provide richer experiences for those who prefer tactile over visual perception, allowing them to touch the artwork while viewing it and, at the same time, being inclusive for those who cannot see (e.g., the blind and visually impaired). Studying these methodologies and applying them in art could offer useful insights for creating personalized paintings. For instance, it might be possible to personalize sonification algorithms by matching musical elements with visual or kinetic elements to create multisensory experiences based on identifying each observer’s unisensory preferences. These acoustic experiences can also be provided through appropriately positioned speakers in space, allowing artists to create sound works that produce a spatial perception of sound connected to the painting.

Specifically regarding the detection of body movement during drawing and painting, while it is possible to precisely capture the artist’s movements, enabling an accurate digital representation of artistic strokes and gestures, the models developed in computational neuroscience to describe how our body’s movements are generated have demonstrated certain specificities of so-called “biological movement,” which the artist certainly must, consciously or unconsciously, consider. For example, the relationship between hand movement speed and the curvature of the trajectory during movement (the greater the speed, the less curvature) (Lacquaniti et al., 1983; Flash and Richardson, 2002), or the speed variation during a hand movement approaching an object (Morasso, 1981). These and other findings show that the “body schema” (Morasso et al., 2015) undoubtedly plays a fundamental role in imagining and executing “artistic action.” The relationship between speed and trajectory of movement and the feedback from force and tactile sensation stimulated by the contact between the held tool and the canvas transforms, through this motor schema, the imagined stroke into a permanent one. The limits imposed on movement by our physical body and the architecture of our nervous system have been studied both concerning “whole body” movement and in the case of precision movements like handwriting (Morasso and Mussa Ivaldi, 1982; Palmis et al., 2017), highlighting peculiarities that could add new information when analyzing the creation of artworks and the evolution of an artist. This is particularly relevant considering that motor abilities change with age, emotional state, and/or as a consequence of pathological conditions (can the evolution of Pierre-August Renoir’s rheumatoid arthritis be traced analogously to how Claude Monet’s cataract progression was visually highlighted in his work?).

These examples demonstrate how new technologies can help us shed some light on the world of painting, both in its study through neuroscientific approaches and in its production, allowing artists to create engaging and personalized pictorial works that could involve a wide range of human senses.

## 4 Discussion: How can painting, neuroscience, and new technologies interact?

The artistic act is a synergy between technique and creativity, a blend of “athletic” skills and cognitive abilities. On one hand, technique represents motor mastery, the ability to shape painting, colors, and forms with precision and control. It is the expression of the artist’s motor competence. On the other hand, imagination and creativity play an equally crucial role. This artistic dimension emerges from the artist’s mind. It is fueled by experience and knowledge, combined with the unique perspective of the creator. Creativity is the expression of originality, the ability to conceive and communicate something new and meaningful. Unlike technique, creativity is a cognitive ability developed through observing the world, learning, personal reflection, and social interaction. An artist, therefore, must be skilled in both technique and creativity. This means not only being able to perfectly execute artistic motor movements but also the ability to imagine and innovate through the act of painting. Ultimately, art is a field where the painter’s technical knowledge and creative originality intertwine in harmonious communication. Technique without creativity does not produce an “artistic” action, and creativity without technique cannot be fully communicated (it remains in the mind of the imaginer, who becomes an artist only when able to convey it).

In this article, we presented recent advancements in understanding multisensory perception and the association between action and perception in the field of cognitive neuroscience. We also showed how these topics can influence and converge in the analysis of the artistic content of a work, partly thanks to the presence of new and interesting technologies and analysis methodologies and partly due to new knowledge in neuroscience and cognitive and social sciences. In this context, we provided insights into concretely converging these themes. Specifically, we believe that two possible fields of interdisciplinary study could be interesting to explore in the coming years. The first is monitoring interindividual sensory preferences and creating/evaluating multisensory pictorial works that allow a direct link between cognitive neuroscience and painting. The second is a detailed investigation of the mechanisms behind the action of painting creation to build a bridge between gesture, creativity, and perception. We believe these topics could open a new and interesting interdisciplinary field between art, neuroscience, and engineering, potentially leading to new research directions and artistic methodologies aimed at making painting more inclusive and personalized.

To conclude, in the creative process, an initial idea is transformed into something tangible and real. During this process, several elements come into play: perception, which involves multiple senses; action, such as movement; and technology, meaning the tools used that can be technological (e.g., a brush vs. VR). In the article we aim to emphasize that studying the creative process requires taking all these components into account. This is true even though not all of them may be evident in the outcome, for example, the painter’s motion ultimately results in a static image to be viewed, yet the movement itself is essential to the creation.

## Data Availability

The original contributions presented in the study are included in the article/supplementary material, further inquiries can be directed to the corresponding author.
